# A Manual of Infectious Diseases

**Published:** 1897-06

**Authors:** 


**A Manual of Infectious Diseases.** By E. W. Goodall, M.D., and J. W. Washbourn, M.D. Pp. viii., 368. London: H. K. Lewis. 1896.

This book is designed chiefly for the use of students, for whom attendance in the wards of a fever hospital is now compulsory; and as a supplement to bedside teaching we consider, after a careful examination of its contents, that it fulfils its purpose admirably. The clinical descriptions bear evidence that the book is written, as such manuals should be, from actual experience, and the remarks on diagnosis, prognosis, and treatment are really practical and helpful. The sections on etiology, though short, are concise and up to date, and give just the information needed for the student or practitioner interested in the causation and methods of spread of the communicable diseases. The diagrammatic figures showing the distribution of the rashes in the exanthemata are specially valuable for teaching purposes, and help to fix the essential points upon the memory. The temperature charts are well chosen and typical; photomicrographs of bacteria illustrate the chapters on diphtheria, influenza, typhoid, erysipelas, and anthrax; and in the appendix is included a summary of the report of the Medical Superintendents of the Fever Hospitals of the Metropolitan Asylums Board on the use of antitoxic serum.

The student who has read this handy and well-printed manual, while he will not know all there is to know about the communicable diseases, will yet have made a very solid addition to his special medical knowledge, and will have little or nothing to unlearn.

In future editions we should like to see cholera included in the scheme of the book, for the possibility of having to deal with this disease is by no means excluded from the outlook of the student of medicine.

